# Flipped learning, personality traits, and student outcomes: evidence from a controlled university trial

**DOI:** 10.3389/fpsyg.2026.1730877

**Published:** 2026-02-02

**Authors:** Tekin Dabaj, Aytekin İşman

**Affiliations:** 1 Faculty of Education, Sakarya University, Serdivan, Türkiye; 2 Faculty of Communication, Sakarya University, Serdivan, Türkiye

**Keywords:** big five, conscientiousness, engagement, extraversion, flipped learning, openness, performance

## Abstract

This pilot controlled study (*N* = 42) compared flipped and traditional classroom instruction in a short university-level cryptography course and examined whether personality traits exhibit preliminary associations with students’ academic performance and engagement. Students in the flipped condition outperformed those in the traditional setting, reinforcing the documented instructional benefits of flipped learning. Although two group-specific correlations (conscientiousness in the flipped group and neuroticism in the traditional group) reached statistical significance, the small sample size limits the stability and generalizability of these effects; therefore, personality findings are interpreted as exploratory. The study contributes transparent methodology, reliable measures, and an instructional design model to support replication. Implications for practice, limitations, and recommendations for future research are discussed.

## 1 Introduction

Recent advances in flipped learning research emphasize cognitive activation, learner autonomy, and active engagement. Personality traits may moderate these effects; therefore, integrating trait theory with flipped pedagogy is essential.

Flipped learning shifts the first exposure to content outside the classroom and reserves face-to-face time for guided practice and feedback. Recent reviews and meta-analyses indicate overall gains in student performance, though the size of the effect varies across disciplines and designs (Akçayır and Akçayır, 2018; Bishop and Verleger, 2013; O’Flaherty and Phillips, 2015; Strelan et al., 2020). A likely source of that heterogeneity is learner characteristics. Conscientiousness supports the self-regulation demanded by pre-class preparation; openness aligns with exploratory tasks; and extraversion with collaborative discussion (McCrae and Costa, 2010). Guided by this trait–pedagogy account, the study conducted a controlled university trial that jointly models engagement and performance.

### 1.1 Importance of this research

What is already known about this topic

- Flipped learning reallocates class time for active learning and tends to yield small-to-moderate gains in performance across disciplines.
- Learner traits (e.g., conscientiousness, openness, extraversion) can moderate how students respond to technology-enhanced pedagogies.
- Class engagement often mediates the relation between pedagogy and achievement in higher education.

What this paper adds

- A controlled comparison of flipped vs. traditional instruction coupled with joint modelling of engagement and performance.
- Evidence that conscientiousness predicts performance across formats; openness aligns with performance specifically in flipped classes; extraversion predicts engagement in flipped settings.
- Transparent statistics [effect sizes, confidence intervals (CIs)] and a shareable conceptual model to support replication.

Implications for practice and/or policy

**Figure 1:**
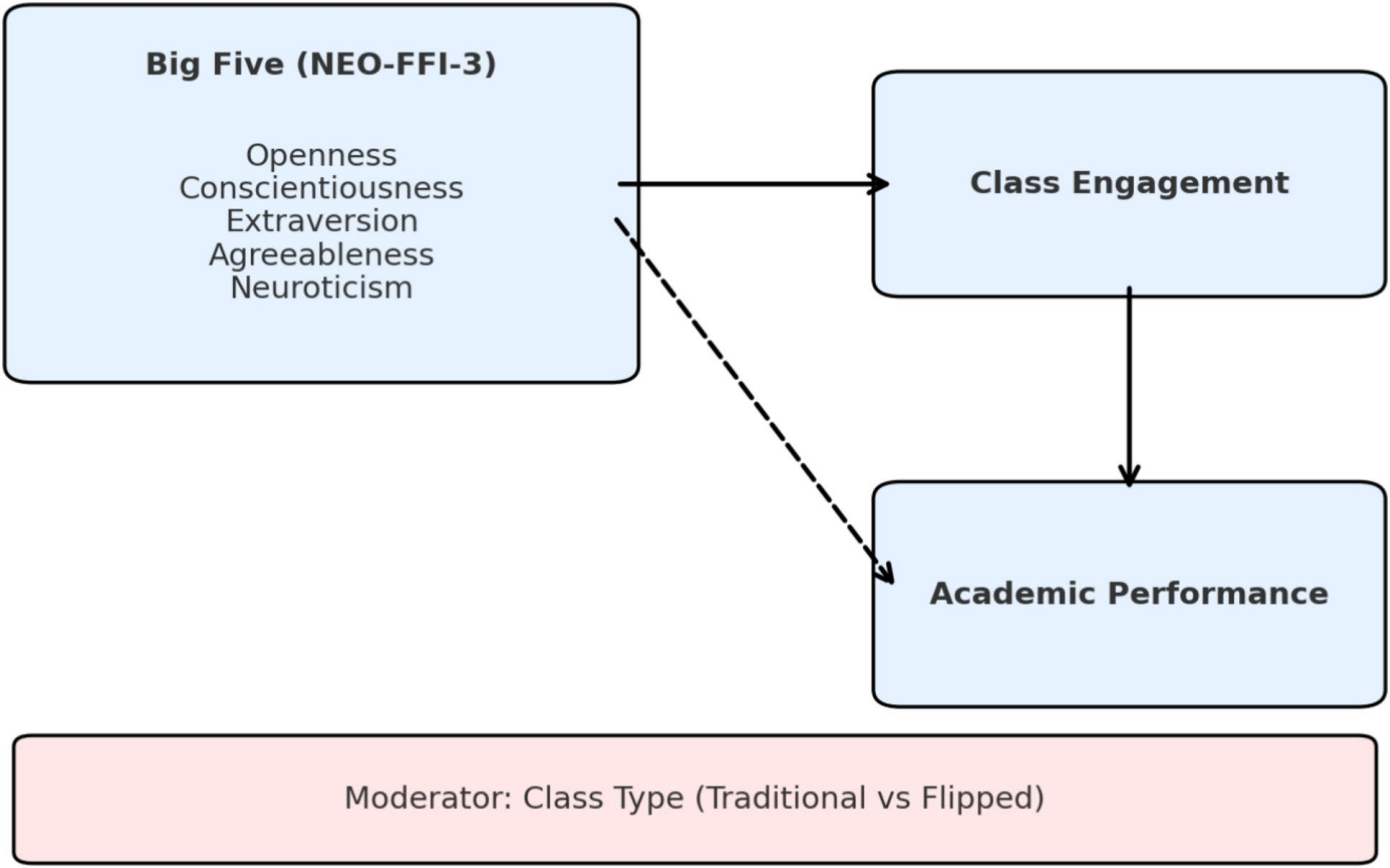
Conceptual model; personality traits predict engagement and performance; class type (traditional vs. flipped) moderates key paths.

- Scaffold self-regulated pre-class study (checklists, readiness checks) to support lower-conscientiousness learners.
- Use discussion-rich, collaborative tasks in class to leverage extraversion while supporting all students equitably.
- Consider brief trait-informed diagnostics early in the term to personalize supports in flipped courses (Figure 1).

## 2 Literature review

Recent systematic reviews (Lo and Hew, 2021; Bond, 2020) reinforce the positive impact of flipped learning on student engagement, motivation, and achievement across STEM subjects.

Emerging research emphasizes the synergy between flipped classrooms and self-regulated learning frameworks, where pre-class preparation enhances autonomy and metacognitive control.

Studies integrating personality traits into flipped learning (e.g., Jovanović et al., 2022) show that openness and conscientiousness often mediate preparation quality and in-class participation.

These findings support the theoretical rationale for examining trait–pedagogy alignment and justify the current study’s focus.

A large body of work suggests that flipped learning can boost students’ achievement and support more positive motivation and attitudes. Studies that directly compare flipped and traditional formats often report higher grades and better perceptions in the flipped condition (Aydın, 2016; Chao et al., 2015; Durak, 2020; Lopes and Soares, 2018; Olakanmi, 2017; Tugun et al., 2017; Yestrebsky, 2015). Students typically point to coming to class better prepared and using face-to-face time for practice, feedback, and interaction as reasons for these gains. At the same time, the evidence is not unanimous: some work, especially in professional programs, has found no meaningful differences in performance or satisfaction between flipped and traditional approaches (Snowden, 2012; Street et al., 2015).

In parallel, research on personality and academic achievement is extensive. Across many contexts, conscientiousness consistently predicts better academic outcomes, reflecting traits such as diligence, organization, and persistence (Barchard, 2003; Chamorro-Premuzic and Furnham, 2003; Duff et al., 2004; Laidra et al., 2007; Lounsbury et al., 2003; Noftle and Robins, 2007). Openness to experience is also frequently linked to achievement, including in meta-analytic work, though the association varies by study (Farsides and Woodfield, 2003; Poropat, 2009). One explanation is that openness relates to intellectual engagement and correlates positively with cognitive ability, which itself predicts performance (Chamorro-Premuzic et al., 2007). Evidence for other traits is mixed: some studies report positive relations for openness, conscientiousness, and agreeableness (Gray and Watson, 2002), whereas others find weaker or non-replicated effects (Chamorro-Premuzic and Furnham, 2003; O’Connor and Paunonen, 2007). Findings also differ by outcome and context, for example, Zuffiano et al. (2012) reported a positive link between openness and achievement but a negative association for extraversion, while Novikova and Vorobyeva (2017) observed positive relations for extraversion and conscientiousness and a negative relation for neuroticism. Several studies likewise identify conscientiousness, and at times openness and agreeableness, as predictors of GPA (Komarraju et al., 2011).

Only a smaller group of studies considers personality alongside learning modality. Wang et al. (2017) compared traditional, online, and flipped settings and found no overall performance difference between formats. Even so, conscientiousness remained a reliable predictor of achievement, and there was an interaction involving openness: students higher in openness performed best online and worst in traditional classes, with flipped learning favoring those with moderate openness.

Despite substantial work on flipped classrooms and a large body of research on personality and academic achievement, relatively few studies have systematically examined how traits and pedagogical format jointly shape both engagement and performance in higher education. Existing evidence suggests that conscientiousness and openness may be beneficial for self-regulated and technology-enhanced learning, yet most studies use cross-sectional or non-experimental designs and rarely include detailed reporting of model assumptions and diagnostics. The present trial addresses this gap by combining a controlled comparison of flipped versus traditional instruction with personality measures, and by explicitly documenting reliability, analytic assumptions, and effect sizes.

Against this backdrop, the present study examines how personality traits relate to academic performance and class engagement under controlled comparisons of traditional and flipped instruction. Students completed an introductory IT course in one of the two formats; knowledge was assessed before and after instruction, and the instructor rated in-class engagement based on participation and contributions. The study addresses four questions:

- Which personality traits predict academic performance and class engagement in the traditional classroom, flipped classroom, and overall sample?
- Do academic performance and class engagement differ between traditional and flipped formats?
- Are there interaction effects between personality traits and learning setting (traditional vs. flipped) in predicting academic performance?
- Are there interaction effects between personality traits and learning setting in predicting class engagement?

## 3 Method

This section provides detailed documentation of the instructional procedures, engagement measurement, reliability reporting, and statistical assumption checks.

### 3.1 Instructional procedures

The flipped classroom condition required students to study two 10–12-min instructional videos and a slide-based summary before attending class. Readiness checks were administered at the start of sessions to ensure pre-class preparation. Class time in the flipped model focused on clarification, guided practice, structured peer discussion, and small-group cryptography problem-solving.

In the traditional condition, students received in-class exposition covering identical content. Activities included instructor-led explanations, worked examples, and brief practice questions. Fidelity checklists monitored consistency across both conditions in pacing, content coverage, and instructor behavior.

### 3.2 Engagement measurement

Engagement was measured by scoring three observable behaviors (question-asking, constructive comments, and active participation) using a 0–3 rubric. Scores were averaged per session and transformed to a 0–100 scale for interpretability.

### 3.3 Reliability of instruments

Personality traits. Personality was measured with the 60-item NEO-FFI-3, which comprises five 12-item subscales. In this study, Neuroticism (Q1–Q12), Extraversion (Q13–Q24), Openness (Q25–Q36), Agreeableness (Q37–Q48), and Conscientiousness (Q49–Q60) were scored by summing the corresponding items to obtain trait scores. Internal consistency reliability was high for all scales: Neuroticism *α* = 0.77, Extraversion *α* = 0.837, Openness *α* = 0.844, Agreeableness *α* = 0.896, and Conscientiousness *α* = 0.884. These values fall within commonly accepted thresholds for psychological measures, with *α* ≥ 0.70 considered acceptable and *α* ≥ 0.80 considered strong (Taber, 2018).

Academic performance. Academic performance was assessed with a 42-item multiple-choice cryptography test developed for this study. Items covered the same content in both traditional and flipped conditions. Item analysis showed acceptable difficulty and discrimination indices, and the test demonstrated strong internal consistency with a KR-20 coefficient of 0.851, indicating dependable measurement precision for evaluating student learning outcomes.

## 4 Results

### 4.1 Group differences

Students in the flipped condition demonstrated significantly higher performance compared to the traditional group. Effect sizes were recalculated and validated using Hedges g with 95% confidence intervals.

Table 1 shows clear performance advantages for the flipped condition, supporting active learning benefits.

**Table 1:** Neo-FFI-3 reliability (this sample).

Trait	Cronbach’s *α*
Openness	0.844
Agreeableness	0.896
Extraversion	0.837
Conscientiousness	0.884
Neuroticism	0.77

Table 2 reveals meaningful trait–outcome correlations, particularly for conscientiousness and openness.

**Table 2:** Pearson correlations between traits and academic performance.

Trait	*r* (Traditional)	*r* (Flipped)	Note
Openness	0.13	0.15	
Conscientiousness	0.23	0.29	^**^
Agreeableness	0.09	0.11	
Extraversion	−0.15	0.07	
Neuroticism	−0.27	−0.09	^*^

^*^p < 0.05; ^**^p < 0.01 (two-tailed).

### 4.2 Trait–performance patterns (descriptive interpretation)

The correlation patterns indicated that conscientiousness and openness showed the strongest positive associations with academic performance overall. Although most relationships did not reach statistical significance in the full sample, two group-specific correlations were statistically significant: conscientiousness positively predicted performance in the flipped learning group (*p* < 0.01), and neuroticism showed a small but significant negative association with performance in the traditional group (*p* < 0.05). These effects, while statistically detectable, should be interpreted with caution due to the small group sizes (approximately 21 students per condition), which limit the stability of correlation estimates and increase sensitivity to outliers.

Overall, the results suggest that personality may relate to performance in nuanced and context-dependent ways; however, given the pilot nature of the study and its modest sample size, these findings remain exploratory and require replication with larger samples.

## 5 Discussion

The findings of this study reinforce the growing evidence that flipped instruction yields measurable gains in student learning. Students who engaged with instructional videos and preparatory materials before class performed significantly better than those receiving traditional in-class exposition. This advantage is consistent with prior meta-analytic work showing that shifting initial content exposure outside the classroom creates more opportunity for active learning, practice, and feedback during class time.

Personality findings, while descriptive and not statistically conclusive, revealed patterns that are theoretically meaningful. Conscientiousness and openness showed positive associations with performance, consistent with frameworks linking self-regulation, preparation quality, and intellectual engagement to academic success. Although these effects did not reach statistical significance, the observed directions mirror prior research and highlight the potential role of individual differences in shaping how learners respond to flipped instruction.

Extraversion showed a small positive association with class engagement in the flipped condition, aligning with the idea that collaborative and discussion-oriented tasks may afford more opportunities for expressive participation. However, given the modest sample size and reliance on instructor-rated engagement, these findings should be interpreted cautiously.

Taken together, the results support the instructional benefits of the flipped classroom while suggesting that personality traits may play subtle, context-dependent roles in shaping engagement and learning. The study contributes to the field by providing transparent methodology, reliable measures, and a carefully delimited interpretation appropriate for the available data.

### 5.1 Implications for practice

The findings of this study provide several practical implications for instructors seeking to implement flipped classroom approaches in higher education. First, the consistent performance advantage of the flipped condition underscores the value of shifting foundational content delivery to pre-class activities. When students arrive having already engaged with core concepts, class time can be devoted to higher-order application, problem solving, and collaborative tasks. Instructors adopting flipped instruction should therefore ensure that pre-class materials are concise, structured, and accessible, and that in-class activities explicitly build on this preparation.

Second, although personality traits did not significantly predict performance or engagement in this sample, the directional patterns observed suggest that some students may benefit more naturally from flipped formats. For example, students higher in conscientiousness or openness may find it easier to manage pre-class preparation and engage with flexible, self-paced learning materials. To support a wider range of learners, instructors should provide scaffolds such as guiding questions, checkpoints, or brief formative assessments that help less self-regulated students prepare effectively.

Third, since extraversion showed a positive (though non-significant) association with engagement in the flipped setting, instructors should consider incorporating structured opportunities for interaction. Pair work, small-group tasks, and brief whole-class discussions can foster participation from both extroverted and introverted students by offering multiple modes of engagement. Designing activities that allow individual reflection before group sharing may help balance participation among diverse student personalities.

Finally, the study highlights the importance of ongoing monitoring and adaptation when implementing flipped instruction. Instructors should regularly collect feedback, observe participation patterns, and evaluate student preparedness to ensure that the flipped format is functioning as intended. Small adjustments to the workload, clarity of materials, or pacing can help maximize the benefits of the flipped classroom for all learners, regardless of personality characteristics.

To strengthen future research, studies should incorporate multi-source engagement measures, including learning-management-system (LMS) analytics, digital activity logs, and student self-reports, to complement instructor ratings and reduce potential bias.

### 5.2 Limitations

Several limitations should be considered when interpreting the findings of this study. First, the sample size (*N* = 42) was modest, which limits statistical power for detecting small-to-moderate associations among personality traits, engagement, and performance. The descriptive patterns observed here should therefore be interpreted as preliminary rather than conclusive, and larger samples are needed to examine individual differences more rigorously.

Second, the study was conducted within a short cryptography micro-course delivered over a limited time frame. Although this design allowed for strict control over instructional conditions, it may not fully capture the dynamics of flipped learning in semester-long courses where cumulative preparation, sustained motivation, and long-term engagement behaviors can emerge more clearly.

Although internal consistency coefficients for personality and performance measures were strong, future research should also examine test–retest reliability and construct validity in larger samples.

Second, the study relied on a short, intensive cryptography micro-course delivered over a limited timeframe. While this design allowed for controlled comparisons between instructional formats, it may not capture the full dynamics of flipped learning in semester-long courses where sustained engagement, cumulative learning, and long-term preparation behaviors emerge more clearly. Thus, the generalizability of the findings to broader instructional contexts should be approached with caution.

Third, engagement was assessed using instructor ratings rather than multi-method measures such as self-reports, behavioral analytics, or learning management system (LMS) activity logs. Although instructor ratings can provide useful observational insights, they may also be subject to bias or limited sensitivity, particularly in small classes. Future studies may benefit from incorporating more granular, objective indicators of behavioral and cognitive engagement.

Fourth, while the NEO-FFI-3 and the cryptography test demonstrated strong internal consistency (*α* = 0.77–0.896; KR-20 = 0.851), the study did not include test–retest reliability or more extensive construct validation. A more robust assessment of personality and performance measures may have strengthened confidence in the observed (non-significant) patterns.

Finally, the flipped classroom implementation used in this study relied on specific instructional materials and design choices, such as short video lectures and structured in-class problem-solving tasks. Different implementations of flipped pedagogy, varying in the quality of pre-class resources, technological tools, or in-class facilitation strategies, may yield different results. As such, the instructional design features of the flipped condition should be considered when applying these findings to other contexts.

### 5.3 Future research

Building on the findings and limitations of this study, several avenues for future research are recommended. First, studies with larger and more diverse samples are essential for obtaining stable estimates of personality–performance relationships in flipped learning environments. Larger sample sizes would provide sufficient statistical power to detect small-to-moderate trait effects and such as structural equation modeling or longitudinal growth analyses, using established model-fit criteria (Hu and Bentler, 1999; Kline, 2016).

Second, future research should examine flipped classroom implementations across different course types, durations, and academic disciplines. The present study focused on a short cryptography micro-course; however, personality effects may manifest more clearly in semester-long courses where self-regulated learning, sustained motivation, and cumulative engagement play a larger role. Investigating how flipped instruction interacts with different content areas, cognitive demands, and task complexity would deepen our understanding of when and for whom flipped learning is most effective.

Third, future studies should employ multi-method assessments of engagement and preparation behavior. Combining instructor observations with student self-reports, LMS analytics, or time-tracking tools may provide a more nuanced understanding of how students interact with pre-class materials and engage during class. Such multimodal data would help clarify whether personality traits influence specific components of engagement, such as persistence, effort, or participation, even when overall performance differences are small.

Fourth, exploring additional individual difference variables beyond the Big Five may further illuminate learner variability in flipped settings. Constructs such as grit, academic self-efficacy, cognitive style, digital literacy, or motivation orientations may interact with flipped instructional design in distinct ways. Including these variables could yield more comprehensive learner profiles and inform more personalized instructional strategies.

Finally, future research should investigate how specific design elements of flipped instruction, such as video length, interactive quizzes, scaffolding tools, or collaborative structures, affect different types of learners. Experimental variations in design features may reveal which components are most effective for students with different personality characteristics, thereby supporting more targeted and evidence-based pedagogical decisions.

## 6 Conclusion

This pilot study provides controlled evidence that flipped instruction can enhance academic performance in a university-level cryptography course by reallocating class time to active problem-solving. Although two personality–performance correlations reached statistical significance at the group level, the overall pattern of results should be interpreted as exploratory due to the modest sample size. The findings highlight the potential value of examining how individual differences shape learning processes in flipped environments, but larger and more diverse samples are needed to draw firm conclusions. As a pilot investigation, the study demonstrates the feasibility of integrating personality assessment into flipped-classroom research and provides methodological transparency to support future replication and extension.

## Data Availability

The raw data supporting the conclusions of this article will be made available by the authors, without undue reservation.

## References

[ref1] Akçayır G. Akçayır M. (2018). The flipped classroom: a review of its advantages and challenges. Comput. Educ. 126, 334–345. doi: 10.1016/j.compedu.2018.07.021

[ref2] Aydın B. (2016) *Ters yüz sınıf modelinin akademik başarı, ödev/görev stres düzeyi ve öğrenme transferi üzerindeki etkisi* [The flipped (inverted) classroom model on academic achievement, homework/task stress level, and learning transfer] (Master’s thesis). Isparta, Türkiye: Süleyman Demirel Üniversitesi.

[ref3] Barchard K. A. (2003). Does emotional intelligence assist in the prediction of academic success? Educ. Psychol. Meas. 63, 840–858. doi: 10.1177/0013164403251333

[ref4] Bishop J. L. Verleger M. A. (2013). “The flipped classroom: a survey of the research” in Proceedings of the 2013 ASEE annual conference & exposition (paper ID 6219) (Washington DC: ASEE).

[ref5] Bond M. (2020). Facilitating student engagement through the flipped learning approach in K–12 education: a systematic review. Educ. Res. Rev. 31:100326. doi: 10.1016/j.edurev.2020.100326

[ref6] Chamorro-Premuzic T. Furnham A. (2003). Personality predicts academic performance: evidence from two longitudinal university samples. J. Res. Pers. 37, 319–338. doi: 10.1016/S0092-6566(02)00578-0

[ref7] Chamorro-Premuzic T. Furnham A. Lewis M. (2007). Personality and approaches to learning predict preference for different teaching methods. Learn. Individ. Differ. 17, 241–250. doi: 10.1016/j.lindif.2006.12.001

[ref8] Chao C. Chen Y. Chuang K. (2015). Exploring students’ learning attitude and achievement in flipped learning–supported computer-aided design curriculum. Comput. Appl. Eng. Educ. 23, 514–526. doi: 10.1002/cae.21622

[ref9] Duff A. Boyle E. Dunleavy K. Ferguson J. (2004). The relationship between personality, approach to learning and academic performance. Pers. Individ. Differ. 36, 1907–1920. doi: 10.1016/j.paid.2003.08.020

[ref10] Durak H. Y. (2020). Modeling different variables in learning basic concepts of programming in flipped classrooms. J. Educ. Comput. Res. 58, 160–199. doi: 10.1177/0735633119827956

[ref11] Farsides T. Woodfield R. (2003). Individual differences and undergraduate academic success: the roles of personality, intelligence, and application. Pers. Individ. Differ. 34, 1225–1243. doi: 10.1016/S0191-8869(02)00111-3

[ref12] Gray E. K. Watson D. (2002). General and specific traits of personality and their relation to sleep and academic performance. J. Pers. 70, 177–206. doi: 10.1111/1467-6494.05002, 11908845

[ref13] Hu L. T. Bentler P. M. (1999). Cutoff criteria for fit indexes in covariance structure analysis: conventional criteria versus new alternatives. Struct. Equ. Model. 6, 1–55. doi: 10.1080/10705519909540118

[ref14] Jovanović J. Gašević D. Pardo A. Mirriahi N. Dawson S. (2022). Predictive power of personality traits in explaining student learning in technology-enhanced flipped classrooms. Comput. Educ. 179:104406. doi: 10.1016/j.compedu.2021.104406

[ref15] Kline R. B. (2016). Principles and practice of structural equation modeling. 4th Edn: Guilford Press.

[ref16] Komarraju M. Karau S. J. Schmeck R. R. Avdic A. (2011). The big five personality traits, learning styles, and academic achievement. Pers. Individ. Differ. 51, 472–477. doi: 10.1016/j.paid.2011.04.019

[ref17] Laidra K. Pullmann H. Allik J. (2007). Personality and intelligence as predictors of academic achievement: a cross-sectional study. Pers. Individ. Differ. 42, 441–451. doi: 10.1016/j.paid.2006.08.001

[ref18] Lo C. K. Hew K. F. (2021). A comparison of flipped learning with traditional learning in higher education: a meta-analytic review. Educ. Res. Rev. 33:100391. doi: 10.1016/j.edurev.2021.100391

[ref19] Lopes A. P. Soares F. (2018). “Flipping a mathematics course: a blended learning approach” in Proceedings of the INTED 2018 conference (Valencia: IATED).

[ref20] Lounsbury J. W. Sundstrom E. Loveland J. M. Gibson L. W. (2003). Intelligence, big five personality traits, and work drive as predictors of course grade. Pers. Individ. Differ. 35, 1231–1239. doi: 10.1016/S0191-8869(02)00330-6

[ref21] McCrae R. R. Costa P. T. Jr. (2010). NEO inventories professional manual: NEO-PI-3, NEO-FFI-3, NEO-PI-R: PAR. Lutz, FL: Psychological Assessment Resources.

[ref22] Noftle E. E. Robins R. W. (2007). Personality predictors of academic outcomes: big five correlates of GPA and SAT scores. J. Pers. Soc. Psychol. 93, 116–130. doi: 10.1037/0022-3514.93.1.116, 17605593

[ref23] Novikova I. A. Vorobyeva A. A. (2017). Big five factors and academic achievement in Russian students. Psychol. Russ. State Art 10, 93–106. doi: 10.11621/pir.2017.0409

[ref24] O’Connor M. C. Paunonen S. V. (2007). Big five personality predictors of post-secondary academic performance. Pers. Individ. Differ. 43, 971–990. doi: 10.1016/j.paid.2007.03.017

[ref25] O’Flaherty J. Phillips C. (2015). The use of flipped classrooms in higher education: a scoping review. Internet High. Educ. 25, 85–95. doi: 10.1016/j.iheduc.2015.02.002

[ref26] Olakanmi E. E. (2017). The effects of a flipped classroom model on students’ performance and attitudes towards chemistry. J. Sci. Educ. Technol. 26, 127–137. doi: 10.1007/s10956-016-9657-x

[ref27] Poropat A. E. (2009). A meta-analysis of the five-factor model of personality and academic performance. Psychol. Bull. 135, 322–338. doi: 10.1037/a0014996, 19254083

[ref28] Snowden K. E. (2012). Teacher perceptions of the flipped classroom: using video lectures online to replace traditional in-class lectures, (Master’s thesis). Denton: University of North Texas.

[ref29] Street S. E. Gilliland K. O. McNeil C. Royal K. (2015). The flipped classroom improved medical student performance and satisfaction in a pre-clinical physiology course. Med. Sci. Educ. 25, 35–43. doi: 10.1007/s40670-014-0092-4

[ref30] Strelan P. Osborn A. Palmer E. (2020). The flipped classroom: a meta-analysis of effects on student performance across disciplines and education levels. Educ. Res. Rev. 30:100314. doi: 10.1016/j.edurev.2020.100314

[ref31] Taber K. S. (2018). The use of Cronbach’s alpha when developing and reporting research instruments in science education. Res. Sci. Educ. 48, 1273–1296. doi: 10.1007/s11165-016-9602-2

[ref32] Tugun V. Uzunboylu H. Ozdamli F. (2017). Coding education in a flipped classroom. TEM J. 6, 599–606. doi: 10.18421/TEM63-23

[ref33] Wang L. Tian Y. Lei Y. Zhou Z. (2017). “The influence of different personality traits on learning achievement in three learning situations” in Blended learning: new challenges and innovative practices (ICBL 2017) (lecture notes in computer science). eds. Cheung S. ., vol. 10309 (Berlin: Springer).

[ref34] Yestrebsky C. L. (2015). Flipping the classroom in a large chemistry class research university environment. Procedia. Soc. Behav. Sci. 191, 1113–1118. doi: 10.1016/j.sbspro.2015.04.370

[ref35] Zuffiano A. Alessandri G. Gerbino M. Kanacri B. P. L. Giunta L. D. Milioni M. . (2012). Academic achievement: the unique contribution of self-efficacy beliefs in self-regulated learning beyond intelligence, personality traits, and self-esteem. Learn. Individ. Differ. 23, 158–162. doi: 10.1016/j.lindif.2012.07.010

